# Nested‐association mapping (NAM)‐based genetic dissection uncovers candidate genes for seed and pod weights in peanut (*Arachis hypogaea*)

**DOI:** 10.1111/pbi.13311

**Published:** 2019-12-25

**Authors:** Sunil S. Gangurde, Hui Wang, Shasidhar Yaduru, Manish K. Pandey, Jake C. Fountain, Ye Chu, Thomas Isleib, C. Corley Holbrook, Alencar Xavier, Albert K. Culbreath, Peggy Ozias‐Akins, Rajeev K. Varshney, Baozhu Guo

**Affiliations:** ^1^ International Crops Research Institute for the Semi‐Arid Tropics (ICRISAT) Hyderabad India; ^2^ Crop Protection and Management Research Unit USDA‐ARS Tifton GA USA; ^3^ Department of Plant Pathology University of Georgia Tifton GA USA; ^4^ Horticulture Department University of Georgia Tifton GA USA; ^5^ Department of Crop and Soil Sciences North Carolina State University Raleigh NC USA; ^6^ Crop Genetics and Breeding Research Unit USDA‐ARS Tifton GA USA; ^7^ Biostatistics Corteva Agrisciences Johnston IA USA

**Keywords:** nested‐association mapping, pod weight, seed weight, association mapping, linkage mapping, candidate genes, peanut

## Abstract

Multiparental genetic mapping populations such as nested‐association mapping (NAM) have great potential for investigating quantitative traits and associated genomic regions leading to rapid discovery of candidate genes and markers. To demonstrate the utility and power of this approach, two NAM populations, NAM_Tifrunner and NAM_Florida‐07, were used for dissecting genetic control of 100‐pod weight (PW) and 100‐seed weight (SW) in peanut. Two high‐density SNP‐based genetic maps were constructed with 3341 loci and 2668 loci for NAM_Tifrunner and NAM_Florida‐07, respectively. The quantitative trait locus (QTL) analysis identified 12 and 8 major effect QTLs for PW and SW, respectively, in NAM_Tifrunner, and 13 and 11 major effect QTLs for PW and SW, respectively, in NAM_Florida‐07. Most of the QTLs associated with PW and SW were mapped on the chromosomes A05, A06, B05 and B06. A genomewide association study (GWAS) analysis identified 19 and 28 highly significant SNP–trait associations (STAs) in NAM_Tifrunner and 11 and 17 STAs in NAM_Florida‐07 for PW and SW, respectively. These significant STAs were co‐localized, suggesting that PW and SW are co‐regulated by several candidate genes identified on chromosomes A05, A06, B05, and B06. This study demonstrates the utility of NAM population for genetic dissection of complex traits and performing high‐resolution trait mapping in peanut.

## Introduction

Peanut (*Arachis hypogaea* L.) is a cash crop with high market and nutritional values. The major focus of breeding is to increase the yield, which is directly proportional to the number of pods per plant, pod weight and seed weight (Gomes and Lopez, 2005). Preferences related to traits such as oil contents, oleic acid contents, relatively large seed size and testa colour drive demand from industries and consumers ensuring higher prices in national and international markets (Gangurde *et al.*, 2019; Venuprasad *et al.*, 2011). Earlier reports on correlation between seed mass, oil and protein contents showed linear increases in oil and protein contents with increased seed mass (Dwivedi *et al.*, 1990). Significant variation is available in the cultivated gene pool for seed weight, and several conventional breeding programs are also targeting for large‐seeded peanut (Venuprasad *et al.*, 2011). Some earlier reports on the inheritance of pod and seed size in peanut showed that large pod and seed size were dominant to small pod and seed (Balaiah *et al.*, 1977; Layrisse *et al.*, 1980), while other studies reported small pods to be dominant over large pods (Gibori *et al.*, 1978). Seed size also had been reported to be controlled by a single gene (Balaiah *et al.*, 1977), three genes (Pattanashetti *et al.*, 2008) or five genes (Martin, 1967). Others suggested quantitative inheritance of seed weight with additive gene action (Garet, 1976), epistatic effects (Upadhyaya *et al.*, 1992) or maternal inheritance (Hariprasanna *et al.*, 2008).

Quantitative trait locus (QTL) mapping studies have been used in peanut for genetic dissection of complex traits, mainly based on biparental populations (Guo *et al.*, 2016; Kumar *et al.*, 2019; Pandey *et al.*, 2017a, 2017b; Wang *et al.*, 2017), including peanut pod size and weight (Chavarro *et al.*, 2019; Hake *et al.*, 2017; Luo *et al.*, 2017). Multiparental mapping populations or next‐generation mapping populations, such as NAM (nested‐association mapping) and MAGIC (Multi‐parent Advanced Generation Inter‐Cross), have already shown their potential in maize (Yu *et al.*, 2008), wheat (Mackay *et al.*, 2014) and soybean (Xavier *et al.*, 2015). Multiparent populations have advantages over biparental populations as they produce additional recombination breakpoints and increase the allelic diversity and power of QTL detection (Yu *et al.*, 2008). Availability of a high‐density genotyping platform with uniformly distributed genomewide genetic markers is critical for high‐resolution genetic dissection of complex traits and tracking the favourable alleles in a breeding population (Pandey *et al.*, 2012; Pandey *et al.*, 2016; Varshney *et al.*, 2013). Reference genome sequences of both wild diploid progenitors *A. ipaensis* and *A. duranensis* (Bertioli *et al.*, 2016; Chen *et al.*, 2016; Lu *et al.*, 2018), as well as allotetraploid cultivated peanut *A. hypogaea* (Bertioli *et al.*, 2019; Chen *et al.*, 2019; Zhuang *et al.*, 2019), have recently been assembled by the international peanut community and are important resources for sequence‐based trait mapping and candidate gene discovery. This has also facilitated the development of high‐resolution SNP arrays in peanut (Clevenger *et al.*, 2017; Pandey *et al.*, 2017b), which have shown great utility in fine trait mapping in other crops such as rice (Thomson *et al.*, 2017), soybean (Xavier *et al.*, 2018), maize (Yan *et al.*, 2010), wheat (Wang *et al.*, 2014), chickpea (Roorkiwal *et al.*, 2018) and pigeonpea (Saxena *et al.*, 2018; Yadav *et al.*, 2019).

Several years ago, U.S. peanut research community developed two NAM mapping populations with two common parents (Tifrunner and Florida‐07) and eight diverse, unique parents, resulting in 16 biparental recombinant inbred line (RIL) families in order to maximize genetic diversity while meeting practical breeding objectives (Holbrook *et al.*, 2013). The primary objective of this developed genetic resource was to share these populations with the peanut research community and to undertake high‐resolution phenotyping of these populations (Chu *et al.*, 2018; Holbrook *et al.*, 2013). The parents represent a wide range of agronomic, morphological and disease resistance traits, and some biparental populations have been studied for resistance to early and late leaf spot diseases (Chu *et al.*, 2019; Clevenger *et al.*, 2018). These two NAM populations thus could combine the strengths of both linkage and association mapping since the NAM populations have higher power QTL detection as compared with biparental mapping populations (Guo *et al.*, 2016; Wang *et al.*, 2017; Yu *et al.*, 2008). Most importantly, this combination of power and resolution could resolve associations down to the gene level in identified genomic regions. Using a subset of Set A of this collection (Holbrook *et al.*, 2013) which was only available at that time, we assembled two NAM populations with a 2 × 4 design, NAM_Tiftunner (581 lines) and NAM_Florida‐07 (496 lines), to demonstrate both utility and power of the NAM approach for trait dissection of 100‐pod weight (PW) and 100 seed weight (SW) in peanut. These populations were genotyped using the Axiom_*Arachis* 58K SNP array (Clevenger *et al.*, 2017; Pandey *et al.*, 2017b) and phenotyped for 2 years of 2015 and 2016, followed by QTL linkage mapping and genomewide association study (GWAS). This report demonstrates the utility and power of the NAM approach in peanut by producing a high‐density genetic map and identifying QTLs and SNP–trait associations (STAs) with greater significance than those observed in biparental populations (Chavarro *et al.*, 2019; Hake *et al.*, 2017; Luo *et al.*, 2017) in pod and seed weights. These identified markers and candidate genes shed light on potential mechanisms controlling pod and seed development in peanut and may serve as useful markers in molecular breeding programs.

## Results

### Phenotypic variation for pod weight and seed weight in NAM populations

**Figure 1:**
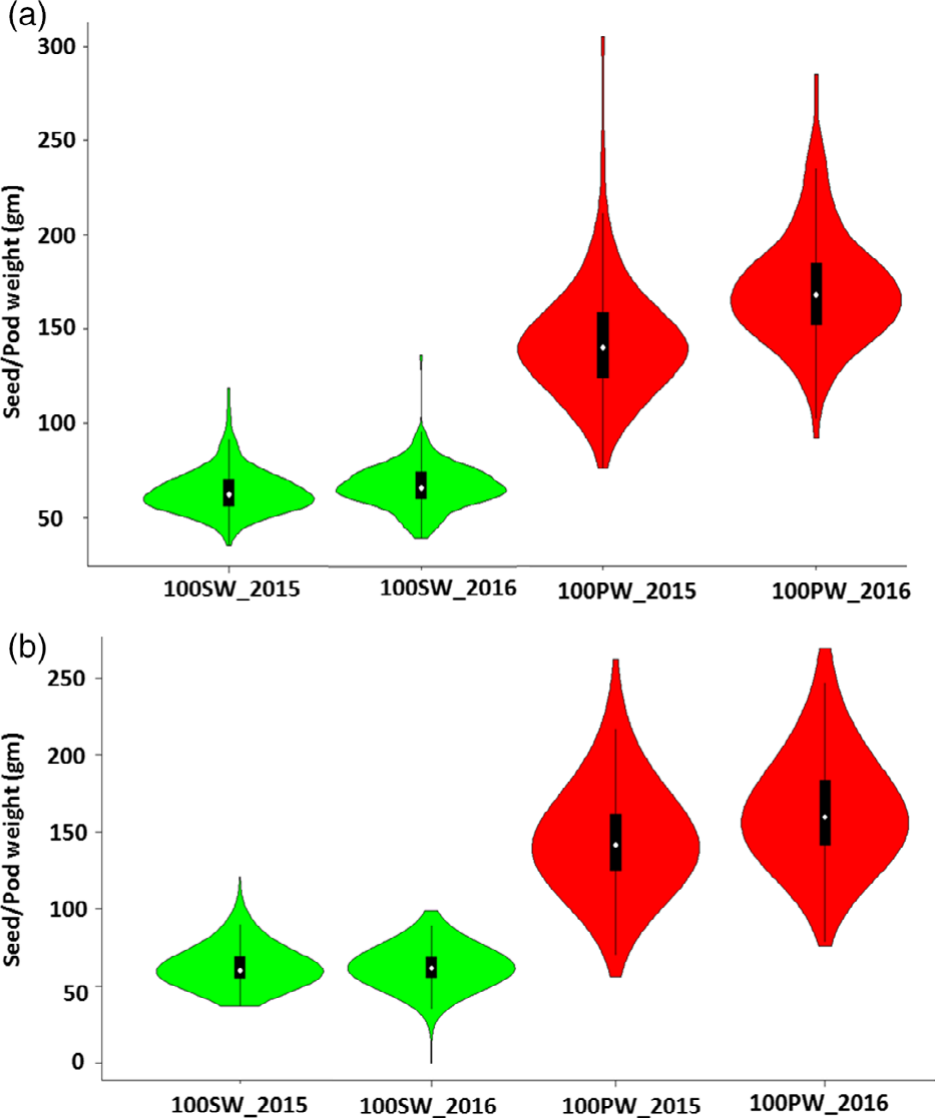
Violin plots represent the variation available in phenotypic data for pod weight and seed weight in nested‐association mapping (NAM) populations. (a) NAM_Tifrunner and (b) NAM_Florida‐07 during season 2015 and 2016.

Significant variation was recorded for 100 pod weight (PW) and 100 seed weight (SW), and the mid‐parental values for PW and SW were close to the population mean. Violin plots showed normal distribution for PW and SW for both populations (Figure 1). Transgressive segregants were observed for PW and SW among the RILs, indicating multigenic inheritance of the traits. There were significant positive correlations between pod weight and seed weight in all two years environments. Little variation was observed between the seasons for PW and SW (Figure 1a, 1B; Tables S1 and S2; Figure S1).

### High‐density genetic maps for NAM populations

**Figure 2:**
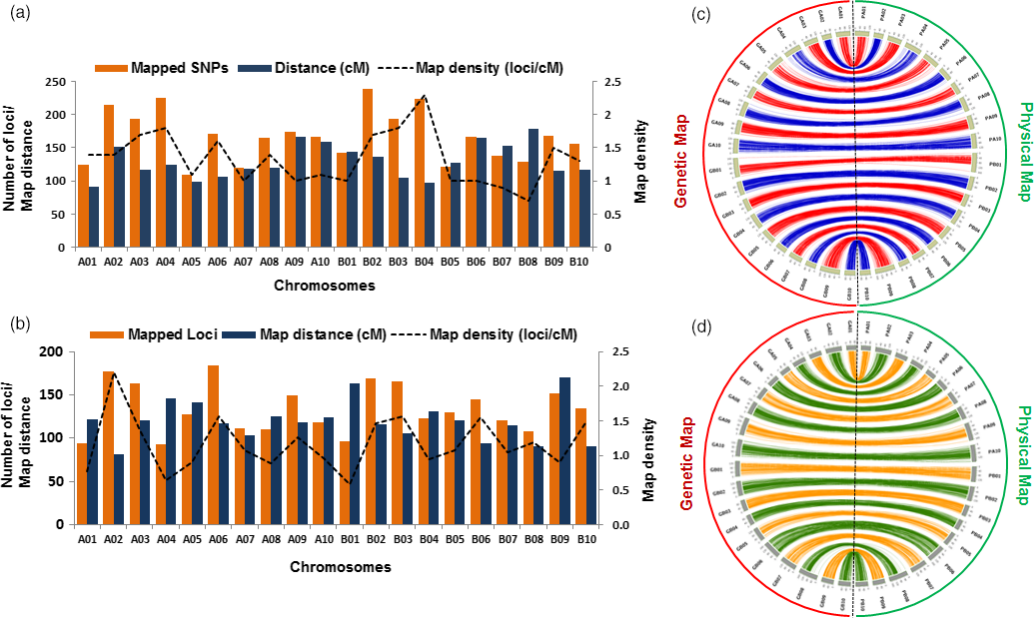
Summary of genetic maps. (a) NAM_Tifrunner, (b) NAM_Florida‐07 with mapped loci, map distance (cM) and map density (loci/cM), (c) collinearity of genetic map with reference genomes (A and B subgenomes) using NAM_Tifrunner population. (d) Collinearity of genetic maps with reference genomes (A and B subgenome) constructed using NAM_Florida‐07 population. The prefix G stands for genetic map on left‐hand side whereas, prefix P stands for corresponding physical map on right‐hand side.

A total of 3874 polymorphic SNPs were used in genetic map construction in NAM‐T. A genetic map was constructed with a total of 3311 polymorphic SNPs spanning 20 linkage groups (Figure 2a). This genetic map achieved a distance of 2,585.9 cM with a map density of 0.77 cM/locus. A total of 1663 and 1678 SNP loci were mapped to the A‐ and B‐ subgenomes, covering 1249 cM and 1336 cM, respectively. The A‐ and B‐ subgenomes achieved a map density of 0.75 and 0.79 cM/locus. The number of mapped loci ranged from 109 on A01 to 238 on B02, while the length of the LGs ranged from 90 cM for A01 to 225 cM for A04. B04 was the densest linkage group with 224 SNP loci mapped achieving a map density of 2.3 loci/cM.

Similarly, a total of 2860 poly‐high‐resolution SNPs were used for construction of a dense genetic map for NAM‐F. A dense genetic map was constructed with 2668 SNPs with a map distance of 2393.4 cM and marker density of 1.1 SNP/cM (Figure 2b). There were 192 SNPs not considered for linkage analysis because of segregation distortion or lack of linkage. A total of 1 326 SNP loci were mapped in the A subgenome spanning 1 197.1 cM, whereas 1 342 SNP loci were mapped in the B subgenome spanning 1 196.3 cM distance. The marker density in both subgenomes was 1.1 loci/cM. The lowest numbers of SNPs were mapped on A04 (93 SNP loci) with the lowest marker density of 0.64 SNP loci/cM. The highest numbers of SNPs (184 SNP loci) were mapped on A06 with a marker density 1.57 SNP loci/cM. A02 had 177 SNPs mapped but had the highest marker density of 2.19 SNPs/cM. Mapping statistics for both NAM populations can be found in (Table S3).

### Highly collinear genetic and physical map

Both genetic maps showed good collinearity with the reference genome sequences of progenitors, *A. duranensis* and *A. ipaensis*. Syntenic regions between the genetic maps (cM) and physical maps (Mb) could be clearly observed on circos plots (Figure 2c,d).

### QTLs for pod weight (PW) and seed weight (SW) in NAM‐T and NAM‐F populations

This study revealed several genomic regions using Joint Inclusive Composite Interval Mapping (JICIM) for PW and SW in both NAM populations (Table 1). A total of 19 QTLs for PW and SW were identified in NAM‐T, whereas 23 QTLs for PW and SW were identified in NAM‐F. The majority of the genomic regions with major effects were identified on chromosomes A05 and B05.

**Table 1:** QTLs identified for pod and seed weights in nested‐association mapping (NAM) populations, NAM_Tifrunner (NAM‐T) and NAM_Florida‐07 (NAM‐F)

QTL name	Chr	Year	Position (cM)	Left flanking marker	Right flanking marker	Marker interval (cM)	LOD	PVE (%)
*QTLs identified in NAM‐T*								
100 Pod weight								
*qPW_A05‐1*	A05	2015, 2016	62	Affx‐152071156	Affx‐152081918	3.4	9.4	32.6
*qPW_A05‐2*	A05	2015, 2016	18.1	Affx‐152072578	Affx‐152034828	1.2	9.8	33.3
*qPW_A05‐3*	A05	2015, 2016	25.3	Affx‐152034514	Affx‐152068537	2.1	12.1	30.4
*qPW_A06*	A06	2015	62.1	Affx‐152028854	Affx‐152042541	0.7	4.1	10.6
*qPW_B05*	B05	2015, 2016	53.2	Affx‐152030151	Affx‐152052489	0.6	8	34.3
*qPW_B06‐1*	B06	2015	86.2	Affx‐152074118	Affx‐152039395	5	3.8	22.1
*qPW_B06‐2*	B06	2015	56.3	Affx‐152063867	Affx‐152068866	6.2	6.1	16
*qPW_B07*	B07	2016	23.2	Affx‐152075138	Affx‐152061032	0.8	3.6	16.3
100 Seed weight								
*qSW_A05‐1*	A05	2015, 2016	18.2	Affx‐152072578	Affx‐152034828	1.2	5.7	30.6
*qSW_A05‐2*	A05	2015, 2016	62.6	Affx‐152071156	Affx‐152081918	3.4	5.3	20.1
*qSW_A06‐1*	A06	2016	65.5	Affx‐152028938	Affx‐152030506	0.8	3.7	18.2
*qSW_A06‐2*	A06	2015	55.4	Affx‐152050526	Affx‐152049487	1.4	3.1	11.8
*qSW_A08*	A08	2016	92.5	Affx‐152034595	Affx‐152050655	2.9	3.4	25.3
*qSW_B05*	B05	2015, 2016	53.1	Affx‐152030151	Affx‐152052489	0.6	4.3	26.1
*qSW_B06‐1*	B06	2016	55.8	Affx‐152026905	Affx‐152044809	2.5	5.1	24.4
*qSW_B06‐2*	B06	2015	86.7	Affx‐152074118	Affx‐152040518	1.7	3.8	22.1
*qSW_B07‐1*	B07	2015	43.6	Affx‐152070761	Affx‐152061732	0.9	8.7	25.3
*qSW_B07‐2*	B07	2016	147.2	Affx‐152034806	Affx‐152027501	9.4	10.8	19.8
*qSW_B09*	B09	2015, 2016	61.3	Affx‐152030882	Affx‐152074441	2.8	4.5	19.2
*QTLs identified in NAM‐F*								
100 Pod weight								
*qPW_A05‐3*	A05	2015, 2016	5.2	Affx‐152077044	Affx‐152051452	4.8	4.9	26.1
*qPW_A05‐2*	A05	2015, 2016	140.1	Affx‐152026889	Affx‐152055218	14	4.3	21.8
*qPW_A05‐1*	A05	2015, 2016	16.4	Affx‐152083713	Affx‐152066050	7.5	3.9	27.4
*qPW_A06‐1*	A06	2015	32.5	Affx‐152031222	Affx‐152041766	1.5	4	13
*qPW_A09‐1*	A09	2016	105.6	Affx‐152070384	Affx‐152079906	2.7	5.3	16.3
*qPW_B05‐1*	B05	2015, 2016	88.3	Affx‐152038129	Affx‐152069488	0.9	3.3	27
*qPW_B05‐2*	B05	2015, 2016	57.7	Affx‐152078919	Affx‐152079844	1.8	3.4	31
*qPW_B05‐3*	B05	2015, 2016	56.8	Affx‐152031298	Affx‐152078919	1	3.3	19.4
*qPW_B05‐4*	B05	2015, 2016	22.8	Affx‐152076736	Affx‐152028895	0.7	4.3	22
*qPW_B06‐2*	B06	2016	74.5	Affx‐152080402	Affx‐152041157	0.8	5.3	32.3
*qPW_B06‐1*	B06	2015	16.6	Affx‐152043214	Affx‐152073378	1.4	4.2	14.6
*qPW_B09‐1*	B09	2015, 2016	112.4	Affx‐152065486	Affx‐152081633	1.6	3.8	16.7
100 seed weight								
*qSW_A05‐1*	A05	2015, 2016	11.2	Affx‐152083713	Affx‐152066050	7.5	4.2	28
*qSW_A05‐3*	A05	2015, 2016	139.3	Affx‐152026889	Affx‐152055218	14	7.7	40.3
*qSW_A05‐2*	A05	2015, 2016	2.2	Affx‐152061849	Affx‐152044122	2.3	3.4	17.9
*qSW_A06‐1*	A06	2015	82.3	Affx‐152083869	Affx‐152039817	0.4	4.7	30.7
*qSW_A07‐1*	A07	2016	78.3	Affx‐152042213	Affx‐152081932	1.4	5.8	34
*qSW_B05‐1*	B05	2015, 2016	109.4	Affx‐152041353	Affx‐152079400	0.8	3.4	21.2
*qSW_B05‐2*	B05	2015, 2016	57.7	Affx‐152078919	Affx‐152079844	1.8	4.6	35
*qSW_B05‐3*	B05	2015, 2016	22.8	Affx‐152076736	Affx‐152028895	0.7	9	40
*qSW_B06‐1*	B06	2015	74.6	Affx‐152080402	Affx‐152041157	0.8	4	34.3
*qSW_B06‐2*	B06	2016	50.2	Affx‐152028010	Affx‐152051179	0.4	3.6	35.7
*qSW_B06‐3*	B06	2015	81.3	Affx‐152048400	Affx‐152027597	1.5	7	36.2

LOD, Logarithm of odds; PVE, phenotypic variance explained.

**Figure 3:**
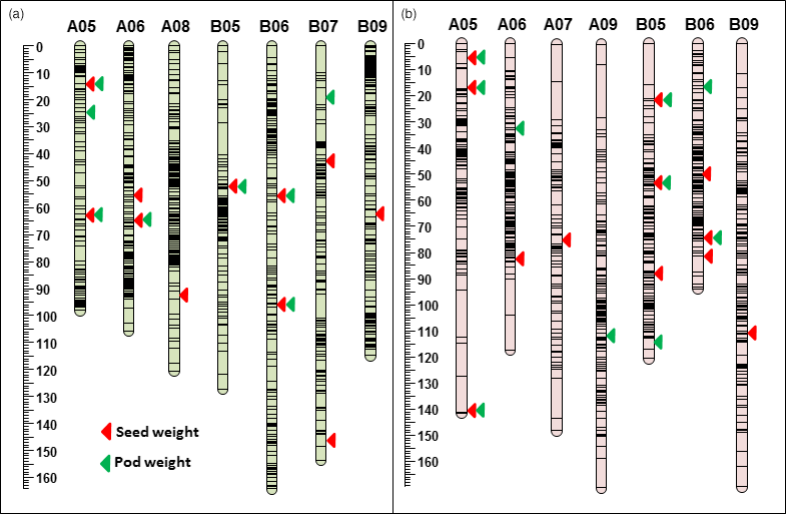
Genomic regions (QTLs) identified for pod weight (PW) and seed weight (SW) in linkage analysis. (a) QTLs identified for pod weight and seed weight using NAM_Tifrunner population. (b) QTLs identified for pod weight and seed weight using NAM_Florida‐07 population. Red triangles for SW and green triangles for PW.

In the NAM‐T, for the trait of PW, there were eight QTLs identified with LOD scores of 3.6 to 12.1 and PVE% of 10.6 to 34.3. The QTL *qPW_B05* identified on chromosome B05 explained the highest PVE of 34.3% with LOD 8.0 for PW. There were two QTLs on chromosome A05 for PW, *qPW_A05‐1* and *qPW_A05‐2,* with over 30% PVE, which also had significant impact on SW (*qSW_A05‐1* and *qSW_A05‐*2), with over 20% PVE (Table 1; Figure 3b). Similarly, for the trait of SW, there were 11 QTLs identified with LOD scores of 3.1 to 10.8 and 11.8 to 30.6 PVE%. The QTL *qSW_A05‐1* which was identified as a major QTL for SW (5.7 LOD and 30.6 PVE %) seems to share same genomic regions where another QTL (*qPW_A05‐2*) was identified for PW (9.8 LOD and 33.3 PVE %). A major QTL for SW was identified on chromosome B09 with 4.5 LOD and 19.2% PVE. One QTL on chromosome A08 was identified for SW with major effect on SW (25.3% PVE). Two QTLs were identified on chromosome B07 showing significant influence on SW. There were genomic regions mostly associated with PW and SW on chromosome A05, B05, A06 and B06 (Table 1; Figure 3b).

In the NAM‐F, 12 QTLs were identified for PW with LOD scores of 3.3 to 5.3 and PVE from 13.0% to 32.3%, including three QTLs on chromosome A05 and four on B05 (Table 1). The highest PVE was recorded for the QTL *qPW_B06‐2* mapped on chromosome B06 at 74.0 cM with LOD 5.3 and 32.3% PVE. Chromosomes A09 and B09 also showed QTLs controlling PW with 16% PVE for each QTL. Similarly, there were 11 QTLs identified for SW with LOD scores of 3.4 to 9.0 and 17.9 to 40.3 PVE%. The QTL *qSW_A05‐3* for SW with the highest PVE was identified on chromosome A05 at 139.0 cM with 7.7 LOD value and 40.3% PVE (Table 1). Similarly, in the B subgenome, a QTL *qSW_B05‐3* was identified on B05 at 22.0 cM for SW with 9 LOD and 39.7 PVE%. Interestingly, five genomic regions were identified as common regions for both PW and SW on chromosomes A05, B05 and B06 (Figure 3a).

### GWAS for pod weight (PW) and seed weight (SW) in NAM‐T and NAM‐F populations

**Figure 4:**
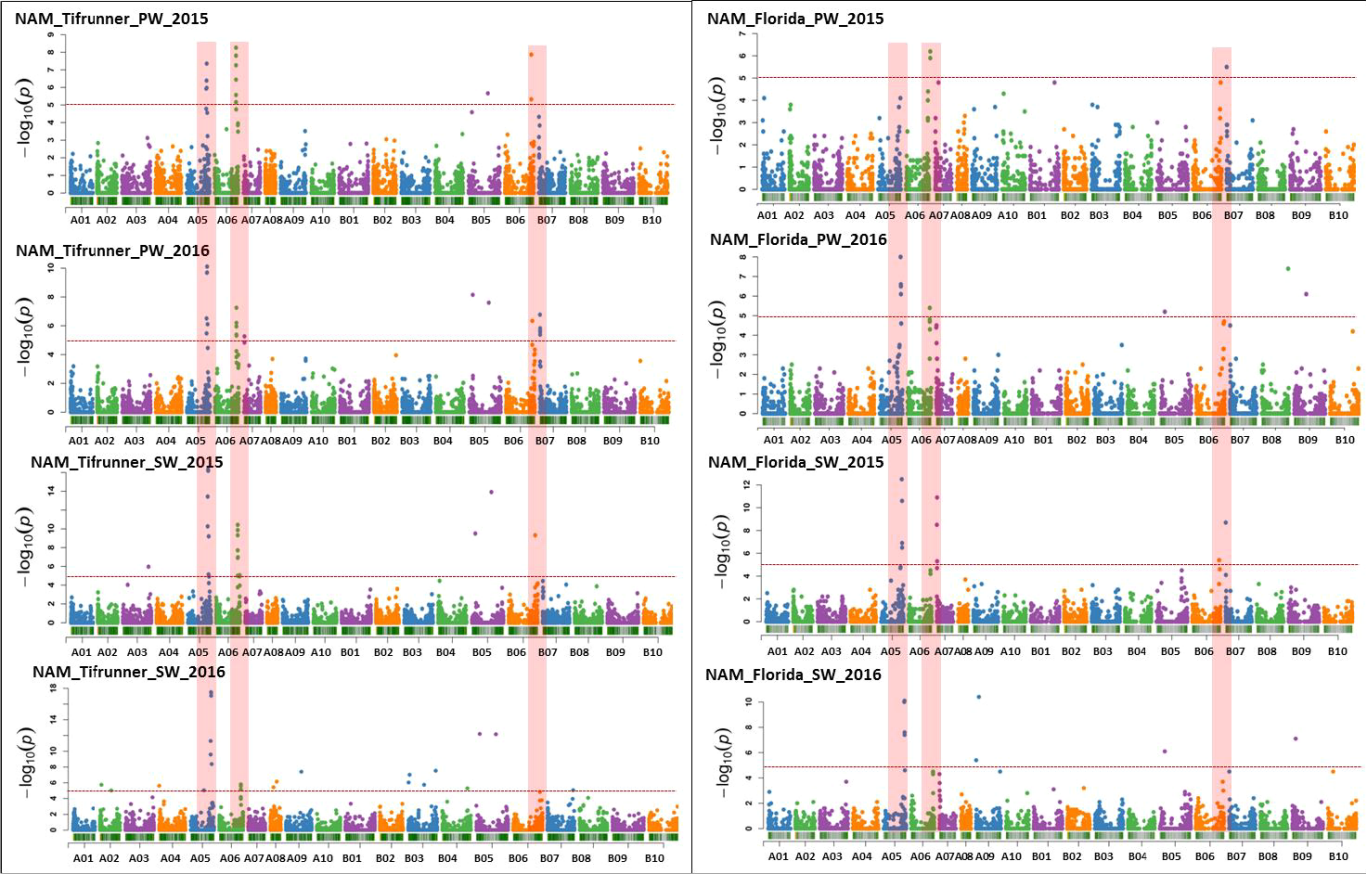
SNP–Trait Associations (STAs) for pod weight (PW) and seed weight (SW) identified using genomewide association study in NAM_Tifrunner and NAM_Florida‐07 populations. Manhattan plots represent the associations for PW and SW. For the NAM‐T, four plots are for PW and SW in two seasons for the years 2015 and 2016. Indicated as NAM_Tifrunner_PW_2015, NAM_Tifrunner_PW_2016, NAM_Tifrunner_SW_2015, NAM_Tifrunner_SW_2016. Similarly, for the NAM‐F, four plots were constructed for PW and SW for two season data during the year 2015 and 2016. Indicated as NAM_Florida_PW_2015, NAM_Florida_PW_2016, NAM‐Florida_SW_2015 and NAM_Florida_SW_2016. The SNPs on chromosomes A05, A06, B06 and B07 highlighted with red colour indicate consistently high association with PW and SW across the seasons.

GWAS results on the NAM‐T population identified 24 potential STAs strongly associated with PW and SW (Table 2). A total of 18 STAs were associated with SW with P value range of 17.5–5.1. All highly associated STAs for SW were identified on chromosomes A05 and B05. Some SNPs were also identified on chromosome A06 and B06 showing minor influence on SW. Additionally, six STAs were identified on A05 and A06 chromosomes with potential candidate genes having reported roles in seed and pod development corresponding to STAs. The SNP on A05 at Affx‐152034807 showed strong association with both PW and SW in all consecutive seasons. Interestingly, all the STAs on chromosomes A05 and A06 were found consistently associated with both SW and PW in both years 2015 and 2016 with very high P values. Similarly, for PW, 20 highly significant STAs were identified with P value range of 17.5–5.3. Almost, all the SNPs identified were associated with both SW and PW on four chromosomes (A05, A06, B05 and B06). Surprisingly, five STAs were identified as unique STAs for just PW on chromosome B07. In the NAM‐T GWAS analysis, 15 SNPs on chromosomes A05, A06, B05 and B06 showed strong and equal association with PW and SW (Table 2; Figure 4; Table S4).

**Table 2:** SNP–trait associations (STAs) and genes corresponding to STAs identified in the NAM_Tifrunner population

SNP	Position in diploid genomes		STA in QTL region	Trait	Year	*P* val	Gene ID	Position of genes in tetraploid genome (AABB)			Function
	Chr	Position						Chr	Start	End	
Affx‐152078443	A05	61633387	*qPW_A05‐1*	SW	2016	9.2	Aradu.398CK	Ahy05	69058788	69056547	Leucine‐rich repeats
Affx‐152026623	A05	93303050	*qPW_A05‐2*	SW, PW	2015, 2016	10.3	Aradu.V498C	Ahy05	99487216	99488936	Nucleoside triphosphatases
Affx‐152044207	A05	93522747	*qPW_A05‐2*	SW, PW	2015, 2016	13.4	Aradu.L6QML	Ahy05	99718318	99718870	myb transcription factor
Affx‐152034807	A05	95201614	*qPW_A05‐2*	SW, PW	2015, 2016	17.5	Aradu.H6YZR	Ahy05	101322384	101321914	Protein kinase superfamily protein
Affx‐152037557	A05	95646799	*qPW_A05‐2*	SW, PW	2015, 2016	17.1	Aradu.5R3CV	Ahy05	101804803	101804290	Unknown protein
Affx‐152068240	A05	95688122	*qPW_A05‐2*	SW, PW	2015, 2016	17.4	Aradu.4D2H2	Ahy05	37914722	37914949	Sphingolipid transporter
Affx‐152060972	A05	97867243	*qPW_A05‐3*	SW, PW	2015, 2016	9.2	Aradu.217QF	Ahy05	104262628	104264458	Pentatricopeptide repeats (PPR)
Affx‐152029724	A06	101049365	*qPW_A06*	SW, PW	2015, 2016	5.4	Aradu.0Y2ZI	Ahy06	106073906	106074903	Concanavalin A‐like lectin/glucanase
Affx‐152072200	A06	101055956	*qPW_A06*	SW, PW	2015, 2016	6.4	Aradu.Z1KSU	Ahy06	106081510	106081280	Unknown protein
Affx‐152042916	A06	101212919	*qPW_A06*	SW, PW	2015, 2016	10.4	Aradu.X5WFU	Ahy06	106322915	106323963	Transcription factor jumonji
Affx‐152034258	A06	101214044	*qPW_A06*	SW, PW	2015, 2016	9.9	Aradu.X5WFU	Ahy06	106322915	106323963	Transcription factor jumonji
Affx‐152044720	A06	101300979	*qPW_A06*	SW, PW	2015, 2016	7.7	Aradu.5LG80	Ahy06	106389203	106388512	Plastid‐lipid‐associated protein (PAP)
Affx‐152041119	A06	101302040	*‐*	SW, PW	2015, 2016	9.3	Aradu.FL7G4	Ahy07	5968474	5968026	Plastid‐lipid‐associated protein (PAP)
Affx‐152058135	A09	71878357	*‐*	SW	2016	7.4	Aradu.20W1Q	Ahy09	72158341	72158836	Basic leucine zipper
Affx‐152072236	B05	17051675	*qPW_B05*	SW, PW	2015, 2016	12.2	Araip.22PIW	Ahy15	17648595	17650297	Acyl‐CoA synthetase
Affx‐152073838	B05	93426656	*qPW_B05*	SW, PW	2015, 2016	13.9	Araip.N2NX2	Ahy15	99201207	99201565	GDP‐fucose protein O fucosyltransferase
Affx‐152075875	B06	125101917	*qPW_B06‐2*	PW	2015	5.3	Araip.YL4Q6	Ahy16	138952178	138951677	Uncharacterized protein
Affx‐152035548	B06	125236516	*qPW_B06‐2*	SW, PW	2015, 2016	9.3	Araip.DII6F	Ahy16	139079635	139080408	Transcription factor jumonji
Affx‐152033888	B07	119939393	*qPW_B07‐2*	SW	2016	5.1	Araip.Z2RR8	Ahy17	128309992	128309051	Protein kinase family protein
Affx‐152067055	B07	1931980	*qPW_B07‐1*	PW	2016	5.6	Araip.H8C7N	Ahy17	2860547	2863439	C2H2‐like zinc finger protein
Affx‐152054860	B07	1941111	*qPW_B07‐1*	PW	2016	5.8	Araip.G1WAG	Ahy17	2870129	2869673	Heat‐shock protein binding
Affx‐152028948	B07	1944622	*qPW_B07‐1*	PW	2016	5.4	Araip.05GH3	Ahy17	2877877	2878795	Mitochondrial transcription termination
Affx‐152043830	B07	1945325	*qPW_B07‐1*	PW	2016	5.7	Araip.R0K9W	Ahy17	2877794	2876876	CCCH‐type zinc finger protein
Affx‐152069626	B07	648238	*‐*	PW	2016	6.8	Araip.Y2DTS	Ahy17	1514508	1513921	Polygalacturonase

In the association panel of NAM‐F, the GWAS results showed a total of 14 STAs significantly associated with PW and SW (Table 3). A total of 10 STAs were found associated with PW located on chromosomes A05, A06, A07, B06, B07 and B09 with p values ranging from 5.4 to 8.7. The SNP Affx‐152042939 was found highly associated with PW with p value of 8.0 and SW with p value of 12.5. A total of 12 STAs were detected for SW. Interestingly, there were eight STAs identified as common STAs for both SW and PW on chromosomes A05, A06, A07, B06 and B07 (Table 3; Figure 4; Figure S2; Table S5).

**Table 3:** SNP–trait associations (STAs) and genes corresponding to STAs identified in the NAM_Florida‐07 population

SNP	Position in diploid genome		STA in QTL region	Trait	Year	*P*‐val	Gene ID	Position of genes in tetraploid genome			Gene features
	Chr	Position						Chr	Start	End	
Affx‐152042939	A05	100238896	*qPW_A05‐2*	SW, PW	2015, 2016	8	Aradu.G6GR7	Ahy05	106199976	106200416	Nodulin MtN21
Affx‐152030262	A05	101618480	*qPW_A05‐2*	SW, PW	2015, 2016	6.6	Aradu.PTC1G	Ahy05	107409300	107409642	Spermidine synthase
Affx‐152073472	A05	101953436	*qPW_A05‐2*	SW, PW	2015, 2016	6.5	Aradu.GEE52	Ahy05	107743343	107743653	Mannose‐1‐phosphate guanylyltransferase
Affx‐152041326	A05	101972210	*qPW_A05‐2*	SW, PW	2015, 2016	6.1	Aradu.VSE1D	Ahy05	107758493	107758020	E2F transcription factor
Affx‐152051216	A06	105402882	*qPW_B06‐2*	PW	2016	5.4	Aradu.U4S3Y	Ahy06	108174915	108174330	ATP‐binding ABC transporter
Affx‐152074153	A07	1191903	*qSW_A07‐1*	SW, PW	2015, 2016	6.2	Aradu.DN3DB	Ahy07	429296	430399	STERILE APETALA
Affx‐152065804	A07	1473208	*qSW_A07‐1*	SW	2015	5.3	Aradu.HR82P	Ahy07	702139	701523	ALG‐2 interacting protein
Affx‐152040866	A07	88041	‐	SW, PW	2015, 2016	5.9	Aradu.P9PXC	Ahy17	1437155	1436877	Rho GDP dissociation inhibitor
Affx‐152032205	A09	13985238	*qPW_A09‐1*	SW	2016	10.4	Aradu.VVP26	Ahy09	14192792	14193209	Aminoacyl‐tRNA ligases
Affx‐152077418	A09	967133	*‐*	SW	2016	5.4	Aradu.N0F41	Ahy09	746523	745565	XH/XS domain‐containing protein
Affx‐152043067	B06	129731047	*qSW_B06‐1*	SW, PW	2015	5.4	Araip.CA56R	Ahy16	146391458	146392020	unknown protein
Affx‐152052942	B07	454008	*‐*	SW, PW	2015, 2016	8.7	Araip.6B9LC	Ahy17	482067	481316	Transmembrane emp24 domain‐containing protein p24beta2‐like
Affx‐152028084	B09	16205446	*qPW_B09‐1*	SW	2016	7.1	Araip.25DGX	Ahy19	16462456	16462950	Helicase‐like protein
Affx‐152036034	B09	57421497	*qPW_B09‐1*	PW	2016	6.1	Araip.M90GE	Ahy19	58395165	58394788	Cytidine/deoxycytidylate deaminase

In the association panel of the NAM‐T, PW‐ and SW‐related genes were identified such as protein kinase superfamily protein, sphingolipid transporter, myb transcription factor, acyl‐CoA synthetase, plastid‐lipid‐associated protein PAP, pentatricopeptide repeat (PPR), sucrose synthase (Table 2). The identified genes are known for crucial role in seed and pod development. In the association panel, NAM‐F (Table 3), nodulin *MtN21* (Aradu.G6GR7), transporters of the amino acid and auxins, showed association with the SNP loci mapped on chromosome A05. This SNP has been potentially associated with PW and SW across the seasons consistently. Spermidine synthase (Aradu.PTC1G) has been reported for its role in embryonic development which was equally associated with PW and SW consistently. E2F transcription factor (Winged helix‐turn‐helix DNA‐binding domain) (Aradu.VSE1D) corresponded to the QTL on chromosome A05 identified for PW and SW. Mannose‐1‐phosphate guanylyltransferase (Aradu.GEE52) relates to the QTL on chromosome A05 has been recorded in two seasons for SW. Acetylglucosaminyl transferase enzyme which is essential for the processing of high‐mannose to hybrid and complex N‐glycans (Araip.SZ4VC) which corresponds to SNP on chromosome B05. Helicases (Araip.25DGX) showed significant association with both SW and PW, which shares the QTL location on chromosome B09. The rho GDP dissociation inhibitor is responsible for root architecture which corresponds to the QTL on chromosome A07. The transmembrane emp24 domain involves in protein trafficking, which relates to the QTL on chromosome B07. Aminoacyl‐tRNA ligases near SNP on chromosome A09 identified for SW.

### Overlapping genomic regions in linkage and association analysis

**Figure 5:**
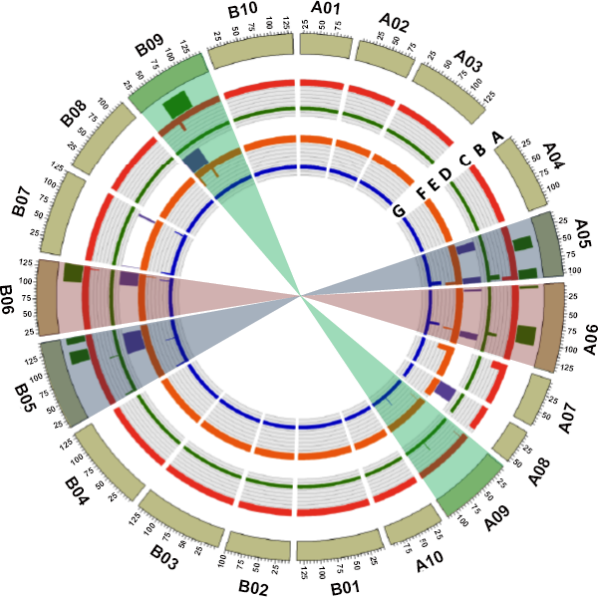
Circos plot represents summary of genomic regions identified in genomewide association study and genetic mapping for pod weight and seed weight in NAM_Tifrunner and NAM_Florida‐07 populations. (a) peanut pseudomolecules from A subgenome are depicted as A01 to A10 and that of B subgenome depicted as B01 to B10; (b) QTLs identified for pod weight and seed weight in NAM_Florida‐07; (c) STAs identified for seed weight in NAM_Florida‐07; (d) STAs identified for pod weight in NAM_Florida‐07; (e) QTLs identified for pod weight and seed weight in NAM_Tifrunner population; (f) STAs identified for seed weight in NAM_Tifrunner; (g) STAs identified for pod weight in NAM_Tifrunner. STAs overlapping in QTL regions were identified on chromosome A05, A06, B05, B06 and B09.

In both NAM populations, co‐localized STAs in QTL regions were identified for PW and SW. In NAM‐T population (Table 2; Figure 5), single STA (Affx‐152078443) was identified in QTL region *qPW_A05‐1.* Five STAs (Affx‐152026623, Affx‐152044207, Affx‐152034807, Affx‐152037557, and Affx‐152068240) with *P*‐value range of 10.3–17.5 were identified for PW and SW located in QTL region *qPW_A05‐2* on chromosome A05. These STAs are detected in both years due to point mutations such as A›G and A›C. One STA (Affx‐152060972) was detected in QTL region *qPW_A05‐3* on chromosome A05. Five STAs (Affx‐152029724, Affx‐152072200, Affx‐152042916, Affx‐152034258 and Affx‐152044720) were detected in QTL region *qPW_A06*. These STAs also were detected in both years for PW and SW and could be caused by the point mutations at A›G and T›G. Two STAs (Affx‐152072236, Affx‐152073838) were identified in QTL region *qPW_B05* on chromosome B05, while four STAs were detected in QTL region *qPW_B07‐1* on chromosome B07 (Table 2). In NAM‐F population (Table 3; Figure 5), four STAs (Affx‐152042939, Affx‐152030262, Affx‐152073472 and Affx‐152041326) were detected in QTL region *qPW_A05‐2*, which all the STAs were associated with PW and SW in both years and could be linked to the point mutation at A›C, T›C and A›G. Two STAs (Affx‐152074153 and Affx‐152065804) were identified in QTL region *qSW_A07‐1*. One STA was identified in QTL region *qSW_B06‐1* on chromosome B06. Two STAs were identified in QTL region *qPW_B09‐1* chromosome B09. Majority of the STAs are possible linked to the A›G transition. These common genomic regions provide more confidence for further gene discovery and fine mapping studies for PW and SW.

## Discussion

Next‐generation mapping populations (such as NAM, MAGIC) allow intensive genome reshuffling making them suitable for high‐resolution mapping due to broad genetic diversity created through high numbers of recombination events. Emerging next‐generation sequencing technologies (NGS) also have accelerated genomic‐assisted breeding by making the discovery of genetic variation more affordable. Peanut is an allotetraploid legume with large genome size (~2.7 Gb) and narrow genetic diversity, which is the bottleneck for dense genetic mapping. SNP arrays and whole‐genome resequencing (WGRS) are the advanced NGS technologies producing maximum data points for high‐density genetic mapping. Both peanut progenitor genome sequences, *A. duranensis* (A genome) and *A. ipaensis* (B genome) (Bertioli *et al.*, 2016; Chen *et al.*, 2016; Lu *et al.*, 2018), are available with annotations. Recently, assemblies of reference genome have also become available for cultivated peanut (Bertioli *et al.*, 2019; Chen *et al.*, 2019; Zhuang *et al.*, 2019) and will increase the efficiency of such studies in the future. In this study, we have used two NAM populations of set A (Chu *et al.*, 2018; Holbrook *et al.*, 2013) and a highly informative ‘Axiom_*Arachis*’ SNP array which was based on two peanut progenitor genome sequences (Chavarro *et al.*, 2019; Clevenger *et al.*, 2017; Pandey *et al.*, 2017b) for genotyping both NAM populations.

### SNP array‐based high‐density genetic maps for multiparent populations

This report demonstrated the advantages of phenotypic analysis of nested‐association mapping (NAM) populations in peanut, combined with genomewide SNP genotyping over the earlier developed SSR‐based genetic maps that were sparse and, therefore, resulted in low genome coverage with possible absence of relevant recombination breakpoints. The fewer recombination events and narrow genetic diversity in biparental RIL populations may result in poor QTL detection power (Gangurde *et al.*, 2019). In this study, we were reporting two dense genetic maps of 3,341 loci for NAM‐T and 2,668 loci for NAM‐F population. The map distance for NAM‐T and NAM‐F was 2586.0 cM and 2393.7 cM, respectively, which are very close to the physical map distance of 2538 Mb (excluding scaffolds) of tetraploid peanut genome (Bertioli *et al.*, 2019). Earlier constructed genetic maps for individual RIL populations were not very dense due to less allelic diversity and few recombination events. Recently, a genetic map of 585 loci using genotyping‐by‐sequencing (GBS) was used for mapping stem rot resistance in peanut (Dodia *et al.*, 2019). A SSR‐based genetic map was developed for mapping aflatoxin resistance with 1219 loci (1175 SSR markers and 42 transposon markers) in 2037.75 cM (Yu *et al.*, 2019). Most of the studies reported the genetic maps between a range of 600–1500 loci in biparental populations, except a WGRS based genetic map with 8869 loci in 3120 cM (Agarwal *et al.*, 2018). In other crops, similar studies using NAM populations successfully dissected the genetics of complex traits and facilitated candidate gene discovery, such as in soybean (Song *et al.*, 2017; Xavier *et al.*, 2018), maize (McMullen *et al.*, 2009; Yu *et al.*, 2008), wheat (Hu *et al.*, 2018; Jordan *et al.*, 2018) and rice (Fragoso *et al.*, 2017). In most of these studies, the GWAS analysis was performed in NAM populations instead of constructing genetic maps. In this study, we constructed a consensus genetic map based on the genotypic data generated from the four families of each NAM population. The genetic map information successfully facilitated QTL discovery in these NAM populations, which provided an opportunity for comparing results with GWAS analysis for SW and PW in peanut.

### Linkage and association analyses uncover candidate genomic regions and genes controlling pod and seed weights

In peanut, the genetic dissection of important traits has been carried out using QTL mapping of segregating RIL populations derived from biparental crosses (Agarwal *et al.*, 2018; Chavarro *et al.*, 2019; Lu *et al.*, 2018; Luo *et al.*, 2018; Pandey *et al.*, 2016). The NAM design has been successful in several crops to exploit the benefits of both joint linkage analysis and association mapping simultaneously in rapeseed and maize (Hu *et al.*, 2018; McMullen *et al.*, 2009) to dissect the genetic basis of complex quantitative traits. In this study, NAM design was used for identification of genomic regions by genetic mapping on two NAM populations, and we performed GWAS by keeping into account the genetic effects produced by each family. The associated SNPs within QTL regions could track the potential genes associated with PW and SW. The traits of PW and SW are the polygenic traits controlled by several genes (Han *et al.*, 2012; Liu *et al.*, 2015). Joint inclusive composite interval linkage mapping identified QTL with major effects for flowering time‐related traits in a maize NAM population (Li *et al.*, 2016) and inflorescence size (Wu *et al.*, 2016). In this study, genomic regions were discovered as co‐located genome regions on chromosomes A05 and B05 controlling both PW and SW. Earlier studies using low‐dense SSR‐based genetic maps reported 14 QTLs with ~17% PVE for PW and SW under drought stress (Ravi *et al.*, 2011; Varshney *et al.*, 2009) leading to identification of small effect QTLs. A genetic mapping study of a RIL population reported three significant QTLs located in a region of 2.7 Mb at the end of chromosome A05 for SW (Luo *et al.*, 2017). In GWAS analysis, five marker–trait associations (MTAs) identified for seed weight using SSRs and DArT loci (Pandey *et al.*, 2014). Recently, a major QTL identified on chromosome A05 for seed number per pod using a biparental cross (Chen *et al.*, 2019). QTL meta‐analysis using consensus map narrowed down the QTL region to 0.7 cM on chromosome A05 (Lu *et al.*, 2018). In this study, seven and four STAs identified in NAM‐T and NAM‐F, respectively, co‐located in QTL regions of PW and SW in both seasons on chromosomes A05. Chu *et al. *(2019) identified a QTL on B05 overlaps for pod yield and LLS resistance. Interestingly, in this study we also reported QTLs for both PW and SW in both NAM populations on B05. STA (Affx‐152030262) corresponds to spermidine synthase (*spds*) on A05 controlling seed size in cereals as reported in rice (Tao *et al.*, 2018). Luo *et al.* (2018) reported SNPs associated with high shelling percentage on chromosome A09 and B02 in peanut. However, this study identified a STA (Affx‐152032205) with a high p‐value (10.0) which was located on chromosome A09 in the vicinity of SNP identified for shelling percentage.

### Candidate genes identified regulating seed and pod weight

In this study, the flanking sequences of the genes were surrounded by significantly associated STAs, which are called as potential candidate genes. Among these genes, we focused only those which are having relevance to the traits of PW and SW from their functional annotations available (://www.peanutbase.org). Direct orthologues of a gene with related function in other species were also taken into consideration.

Few genes identified in this study were reported earlier for their direct role in regulation of SW and PW in other species. The STA (Affx‐152030262) on chromosome A05 corresponding to the spermidine and spermine, which were reported as low molecular organic cations and found in organisms from bacteria to plants and animals (Alcázar *et al.*, 2006). Orthologues of spermidine synthase (*spds*) have been reported to play a role in embryonic development (Yoshihisa *et al.*, 2004). Editing of spermidine synthase using RNAi resulted in malformation of the embryos which affects seed weight in rice (Imai *et al.*, 2004). *Spds* has been reported for its role in regulation of seed size, yield and seed germination (Tao *et al.*, 2018). An E2F factor corresponding to STA (Affx‐152041326) was identified on chromosome A05 which is reported for its major role in cell growth and proliferation as well as in development of the seed coat (Tim *et al.*, 2009). Mannose‐1‐phosphate guanylyltransferase that was flanked by the STA (Affx‐152041326) plays a vital role in plant development and cell‐wall architecture as it mediates N‐linked glycosylation for cellulose biosynthesis (Wolfgang *et al.*, 2001). Cellulose is important component of peanut seed coat and pod shells (Wan *et al.*, 2016); hence, the mannose‐1‐phosphate guanylyltransferase might be involved in the regulation of PW and seed coat of seed. Nodulin was identified on chromosome (A05) flanked by STA (Affx‐152042939) reported to be expressed in root nodules and seed as well as pods (Clevenger *et al.*, 2016). Nodulins have an important role in transporting nutrient, solutes and hormones throughout plant growth and development (Denance *et al.*, 2014). During pod filling, nodulins might be playing a major role for solute transport which may be affecting seed weight and pod weight.

As PW and SW are very complex traits, STAs with small effects were also identified which may involve as activators or enhancers in regulation of important genes (Table 2). Aradu.H6YZR (protein kinase) was reported to be involved in the various biochemical pathways such as nutrient signalling, protein phosphorylation. Two kinases SNRK2.2 and SNRK2.3 regulate abscisic acid (ABA) levels which affects seed germination, dormancy and seedling growth in Arabidopsis (Fujii *et al.*, 2007). Aradu.4D2H2 (sphingolipid) the proteins play a role in the endosome/lysosome storage, signal transduction across the plasma membrane, plasma membrane stability and the structural components of cell wall (Chao *et al.*, 2011). However, sphingolipids are not very closely associated with the pod or seed development. Aradu.L6QML (transcription factor MYB62) plays an important role in various cellular processes such as resistance against biotic, abiotic stresses and developmental processes (Ambawat *et al.*, 2013). MYB89 (R2R3‐MYB transcription factor) highly expresses in developing seed during maturation which acts as a repressor for oil accumulation in seeds. The knockout of MYB89 factor resulted into high oil accumulation in *myb89‐1* mutants in Arabidopsis (Li *et al.*, 2017). Araip.22PIW (acyl‐CoA synthetase) serving as the carbon source for fatty acid biosynthesis in Arabidopsis which triggers oil accumulation in seed therefore might be associated with seed mass (Lin and Oliver, 2008). Transcription factor jumonji is a class of proteins in Arabidopsis reported to be involved in the regulation of flowering with other transcriptional factor (Noh *et al.*, 2004). Knockdown of a jumonji JMJ524 in tomato resulted into shrunken leaves and shortened internodes, but increased levels of gibberellic acid (GA3) reported in mutants (Li *et al.*, 2015). Plastid‐lipid‐associated protein (*PAP*) (Aradu.FL7G4) involved in the sequestration of hydrophobic compounds such as lipids into seed endosperm. PAPs interact with MYB transcription factors during ABA metabolism which mediates signal transduction in response to biotic and abiotic stresses (Leitner‐Dagan *et al.*, 2006). Pentatricopeptide repeats (Aradu.217QF) play role in cellular organelles interactions, organelles biogenesis, photosynthesis and respiration (Barkan and Small, 2014). Sucrose synthase (Aradu.PD37S) plays role in starch and sucrose metabolism, crucial in determining the source and sink loading during transportation of photosynthesis products into seed and pod (Baroja‐Fernandez *et al.*, 2012). Acetylglucosaminyl transferase enzyme has been reported for vitamin C biosynthesis in the plant cell wall (Strasser *et al.*, 2005). Helicases reported for their role in DNA repair and nucleotide metabolisms in plants (Raikwar *et al.*, 2015).

### Two subgenomes share responsibility for pod and seed development in peanut

As the B subgenome (*A. ipaensis*) of cultivated peanut is highly similar to the A subgenome of (*A. duranensis*) (Bertioli *et al.*, 2016), most of the genes have two copies representing their respective genomes. This has resulted in the association of phenotypic data with genomic regions (homologues) from both subgenomes. In support of this, we identified genomic regions on A05/B05, A06/B06, A07/B07 and A09/B09 for SW; also, SNPs for PW were identified on A06/B06 and A07/B07. Interestingly, as the PW and SW are dependent and associated traits, we identified similar candidate genes on chromosomes A06, B06, A07 and B07. The information generated from this study would further help in selecting favourable haplotypes from both the subgenomes to achieve desirable seed and pod features in peanut.

## Conclusion

Until now, only biparental and natural germplasm collections were deployed in peanut for conducting trait mapping and association studies for important traits. This study used a NAM approach using peanut research community developed resource to perform high‐resolution mapping and gene discovery for two important yield‐related traits, that is, seed weight and pod weight. This study also applied the high‐density 58K SNP genotyping assay, Axiom_*Arachis*, which further improved the resolution of trait mapping. Being complex traits, the genetic and GWAS analyses identified potential genomic regions and candidate genes over eight chromosomes (A05, A06, A08, A09, B05, B06, B08 and B09) for seed weight and pod weight. Candidate genes were identified such as spermidine synthase (*spds*), nodulins, pentatricopeptide repeats, E2F and acyl‐CoA synthetases, which may play a significant role in the regulation of pod and seed development and warrant further investigation. The QTLs and STAs identified in this study also serve as a source for potential selectable markers for assistance in molecular breeding selection for new cultivars with desired seed and pod weight for improved yield and the development of lines with seed size specifications meeting the needs of oil, food and confectionary manufacturers.

## Material and methods

### Plant material and phenotyping

Two NAM populations namely ‘NAM_Tifrunner’ (NAM‐T) and ‘NAM_Florida‐07’ (NAM‐F) were defined by using a subset of the Set A (which was only available at that time) RIL populations developed by peanut research community (Chu *et al.*, 2018; Holbrook *et al.*, 2013), two runner cultivars (Tifrunner and Florida‐07) as common parents and four unique parents of N08082olJCT, C76‐16, NC 3033 and GP‐NC WS16 (SPT 06‐06) (Tallury *et al.*, 2014). NAM‐T had 581 RILs and NAM‐F had 496 RILs. NAM‐T has subsets of 161, 162, 132 and 125 RILs and NAM‐F has subsets of 120, 105, 92 and 179 RILs. The subsets of RILs from both NAMs and six parental lines were planted at the USDA‐ARS Belflower Farm, Tifton, GA, for two years (2015 and 2016) for phenotyping of 100‐pod weight (PW) and 100‐seed weight (SW). The NAM lines were planted in two‐row plots (1.5 m long with 90‐cm row space), separated by an alley of 3 m at a seeding rate of six seeds per 0.3 m. Standard agronomical practices for peanut cultivation in Georgia were followed, and no fungicide was applied during the growing seasons. After harvest and drying to less than 10% water content, 100 pods and 100 seeds were picked randomly and weighed for PW and SW traits. Each plot was sampled three times as replications.

### DNA extraction and genotyping with ‘Axiom_*Arachis’* array

DNA samples from all the NAM lines used in this study were extracted from young leaves using Thermo Scientific GeneJET Plant Genomic DNA Purification Mini Kit. The DNA samples were checked for quality on 0.8% agarose gels and quantified on a Nanodrop 8000 Spectrophotometer (Thermo Scientific, Pittsburgh, PA). Affymetrix GeneTitan platform was used to genotype both NAM populations with the 58K SNP ‘Axiom_*Arachis*’ array (Clevenger *et al.*, 2017; Pandey *et al.*, 2017b). Initially, the target probes for 581 samples for NAM‐T and 496 samples for NAM‐F were prepared using a minimum of 20 μL DNA with a concentration 10 ng/μL. The samples were then amplified, fragmented and hybridized on the array chip followed by single‐base extension through DNA ligation and signal amplification according to the procedure explained in the Affymetrix Axiom® 2.0 Assay Manual (http://axiom_2_assay_auto_workflow_user_guide.pdf). The GeneTitan Multi‐Channel Instrument (Affymetrix, Santa Clara, CA, USA) was then used for staining and scanning the samples to derive the genotypic information for each line. The genotypic data for each line were generated and stored in the form of.CEL file format.

### SNP allele calling and quality analysis

The SNP allele calling and data analyses were performed following the process mentioned in Pandey *et al. *(2017b). Initially, the Axiom™ Analysis Suite version 1.0 was used for allele calling by importing.CEL files. Subsequently, we used ‘Best Practices’ workflow to perform quality control (QC) analysis of samples to select only those samples which passed the QC test for further analysis. The ‘Sample QC’ workflow was then used to produce genotype calls for the samples which passed QC analysis using ‘Best Practices Workflow’. The ‘Genotyping’ workflow was used to perform genotyping on the imported.CEL files regardless of the sample QC matrix. Before making the genotyping calls, samples not passing the QC were removed as their inclusion may reduce the quality of the analysed results. Finally, the ‘Summary Only’ workflow was used to produce a summary containing details on the intensities for the probe sets for use in copy number analysis tools. It also allows exporting the SNP data after the analysis are completed for downstream analysis. The above criteria helped in removing the SNPs having low‐quality SNPs and keeping only the poly‐high‐resolution SNPs for the further analysis. The genotyping data from a total of 58 233 SNPs for both NAM populations were retrieved from Axiom analysis suit. The SNP IDs with their corresponding affymetrix IDs and other necessary details are attached in (Table S6). Polymorphic SNPs segregating within each RIL or segregating in at least two RILs were used for genetic map construction. All polymorphic SNPs regardless of segregation distortion with minor allele frequency (MAF = 0.25) and missing threshold (misThr = 0.8) were considered for GWAS. In NAM‐T, 3876 polymorphic SNPs were used for linkage analysis, while a total of 11 520 polymorphic SNPs were used in the GWAS analysis. In NAM‐F, 2860 polymorphic SNPs were used for linkage analysis, while 7672 polymorphic SNPs were used in the GWAS analysis using the R package NAM (Xavier *et al.*, 2015) (Figure S3). Adjacent markers which are 100% identical and carrying similar genotypic values were removed using the parameter perfect symmetry (psy = 1, for 100% symmetry) (Figure S4).

### Construction of dense genetic maps

After filtering the complete genotypic data for the poly‐high‐resolution SNPs, individual SNPs were recoded as ‘B’ representing homozygous for the founder parents (C76‐16, N08082, NC 3033, SPT06‐06) and ‘A’ representing homozygous for common parents (Tifrunner and Florida‐07), ‘H’ representing heterozygous and ‘‐’ representing missing alleles. The genotyping data were first tested for segregation distortion for each SNP marker by a chi‐square test. The genetic map was constructed using JoinMap (v4.0) with LOD score 5.0 and a minimum recombination threshold of 50%. Identical SNP loci and lines were removed using the function ‘exclude identical’. The Kosambi map function was used for genetic map construction and to convert the recombination frequencies into map distances in centiMorgans (cM) (Kosambi, 1944). No attempt has been made to map the distorted SNP loci in the final genetic map. The final chromosome‐wise marker positions with their respective names then used to draw the final genetic map using MapChart (Voorrips, 2002).

### Collinearity of genetic maps of NAM‐T and NAM‐F

Each linkage group in the genetic maps of NAM‐T and NAM‐F was numbered and oriented according to its homologous physical map of diploid candidate genomes *A. ipaensis* and *A. duranensis* (Bertioli *et al.*, 2016). Synteny and collinearity between the maps were visually assessed in circus plot (Krzywinski *et al.*, 2009) by using the position of mapped loci on respective genetic maps (cM) and physical map (bp).

### Joint inclusive composite interval mapping

The genetic map and the phenotypic data were used for QTL analysis. A joint QTL mapping approach across the four families of each NAM‐T and NAM‐F populations was done using the joint inclusive composite interval mapping (JICIM) method implemented in IciMapping 4.1. The JICIM approach is effective and specially designed for joint QTL analysis of NAM populations (Buckler *et al.*, 2009; Li *et al.*, 2011). The genotypic data of both NAMs were recoded, where 0 represents homozygous for founder parent, 2 represents homozygous for common parent, 1 represents heterozygous, and −1 represents missing. QTL analysis was performed using a stepwise regression probability of 0.001. The LOD threshold was calculated by 1000 permutations at the *P* = 0.05 level. QTL effects were estimated as the phenotypic variance explained (PVE) and additive effects by the QTL. Scanning for QTLs was done at an interval of 5 cM, and a QTL was declared significant if the threshold was greater than the 1000 permutation of the trait data by resampling method (Churchill and Doerge, 1994). In JICIM, the additive effect from each family with their respective LOD scores and phenotypic variance were recorded for each QTL.

### Genomewide association study and candidate gene discovery

Genomewide association analyses were performed using the multiparental model, namely mixed linear model (MLM) (Wei and Xu, 2016), implemented in R package for NAM population (Xavier *et al.*, 2015) which followed Equation (1),

(1) y=Xβ+Zα+ψ+ε,

where **y** is the vector of phenotypes, **Xβ** is the design matrix and coefficients of fixed effects, here corresponding to the intercept, **Z** is the incidence matrix of the marker data, **α** is the vector of regression coefficients associated with marker effects within family, **ψ** corresponds to the polygenic coefficients, and **ε** is the vector of residuals. The model assumes that α ~ N (0, **I**σ^2^_α_), **ψ**~N (0, **K**σ^2^_ψ_) and **ε**~N (0, **I** $\sigma_{\varepsilon }^{2}$), where **K** regards kinship among lines framed by the genomic relationship matrix. Statistical significance of single markers was evaluated through the likelihood‐ratio test by comparing the log‐likelihood of the model that includes the marker effect (L_1_) with the log‐likelihood of the model that does not (L_0_). The association threshold to define significantly associated marker with the trait was computed with Bonferroni correction for multiple testing to mitigate false positives. Bonferroni is the most standard procedure, and it is super conservative. Bonferroni threshold (α = 0.05) yielding a threshold of approximately −log10 (0.05/3876) = 4.88 for NAM‐T and −log10 (0.05/2860) = 4.76 for NAM‐F. But, here we used an extra conservative threshold of 5 –log (*P*‐value) for both NAM populations.

The SNPs with significant associations were exploited for candidate gene discovery by using the annotation of diploid genomes, *A. duranensis* and *A. ipaensis* (://peanutbase.org ; Bertioli *et al.*, 2016). The SNP subsiding start and end position of a gene was explored for candidate gene on the basis of their biological function annotation related to the trait of interest. There are possibilities of getting multiple SNPs on a gene segment which can be referred as haplotypes.

## Conflict of interest

The authors declare that there is no conflict of interests.

## Authors’ contribution

SSG, HW, SY, MKP performed data analyses and drafted the manuscript. JCF and AX assisted in data analysis and discussion. YC, CCH and POA developed the populations. HW and AKC assisted in field data collection. RKV and BG designed and finalized the manuscript. BG conceived the project, planned, secured extramural funds, and revised and submitted manuscript.

## Supporting information


**Figure S1** Frequency distribution plots representing the magnitude of phenotypic variation for pod weight (PW) and seed weight (SW) in NAM_Tifrunner and NAM_Florida‐07.

**Figure S2** QQ plots against genotypic and phenotypic data represent the normal distribution for genotypic and phenotypic data.

**Figure S3** Criteria used for filtering the SNPs on the basis of polymorphism and distortion for genetic mapping and genomewide association studies in NAM_Florida‐07 and NAM_Tifrunner population.

**Figure S4** SNP density plots representing** c**hromosomes wise distribution of SNPs used for genome‐wide association studies in (A) NAM_Tifrunner and (B) NAM_Florida‐07 populations.

**Table S1** Phenotypic variability, heritability, skewness, kurtosis for pod weight (PW) and seed weight (SW) in NAM_Tirunner population.

**Table S2** Phenotypic variability, heritability, skewness, kurtosis for pod weight (PW) and seed weight (SW) in NAM_Florida‐07 population.

**Table S3** Summary of genetic map constructed using genotypic data generated using 58K SNP array on NAM_Tifrunner and NAM_Florida‐07 populations.

**Table S4** Summary significantly associated SNPs identified for pod weight (PW) and seed weight (SW) in NAM_Tifrunner population with details of annotation of each gene corresponding to the SNPs and their biological role.

**Table S5** Summary significantly associated SNPs identified for pod weight (PW) and seed weight (SW)in NAM_Florida‐07 population with details of annotation of each gene corresponding to the SNPs and their biological role.

**Table S6** Details of SNPs on 58K ‘Axiom_*Arachis*’ SNP array used for genotyping NAM populations.
