# Supplementary figures and images for: Nested‐association mapping (NAM)‐based genetic dissection uncovers candidate genes for seed and pod weights in peanut (Arachis hypogaea)
Source: Plant Biotechnol J. 2019 Dec 25;18(6):1457–71. doi: 10.1111/pbi.13311 (PMC7206994; doi:10.1111/pbi.13311)

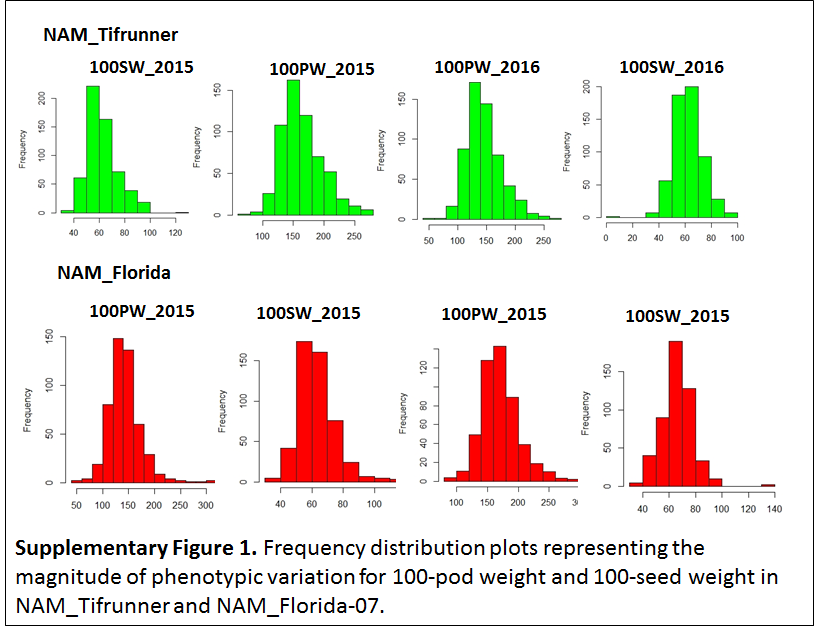

Supplement: Supplementary file 1 — Figure S1 Frequency distribution plots representing the magnitude of phenotypic variation for pod weight (PW) and seed weight (SW) in NAM_Tifrunner and NAM_Florida‐07. [file PBI-18-1457-s001.tif]

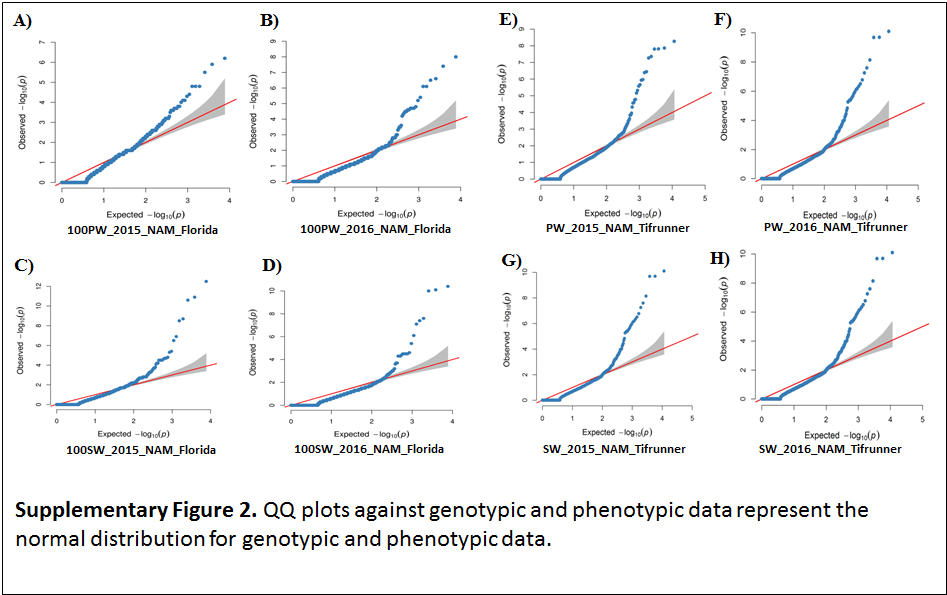

Supplement: Supplementary file 2 — Figure S2 QQ plots against genotypic and phenotypic data represent the normal distribution for genotypic and phenotypic data. [file PBI-18-1457-s002.tif]

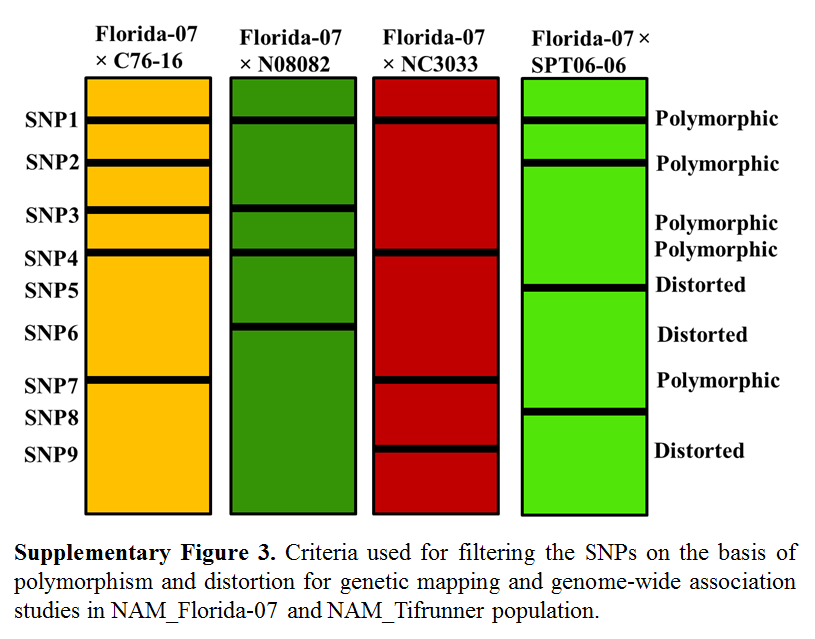

Supplement: Supplementary file 3 — Figure S3 Criteria used for filtering the SNPs on the basis of polymorphism and distortion for genetic mapping and genomewide association studies in NAM_Florida‐07 and NAM_Tifrunner population. [file PBI-18-1457-s003.tif]

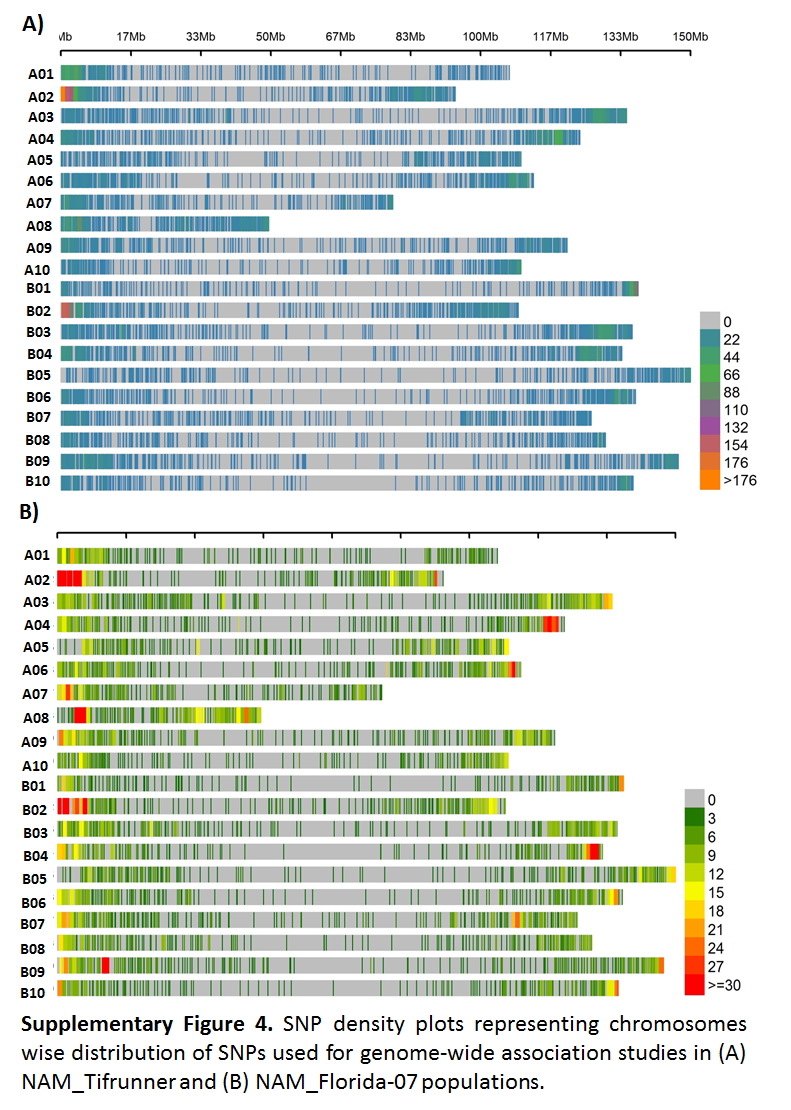

Supplement: Supplementary file 4 — Figure S4 SNP density plots representing chromosomes wise distribution of SNPs used for genome‐wide association studies in (A) NAM_Tifrunner and (B) NAM_Florida‐07 populations. [file PBI-18-1457-s004.tif]
